# Editorial: Pursuing quality education in Physical and Rehabilitation Medicine

**DOI:** 10.3389/fresc.2023.1242522

**Published:** 2023-07-21

**Authors:** Maria Gabriella Ceravolo, Francesca Gimigliano, Jorge Lains

**Affiliations:** ^1^ Department of Experimental and Clinical Medicine, Politecnica Delle Marche University, Ancona, Italy; ^2^ Department of Mental and Physical Health and Preventive Medicine, University of Campania ‘Luigi Vanvitelli’, Naples, Italy; ^3^ Physical Medicine and Rehabilitation, Centro de Medicina de Reabilitação da Região Centro - Rovisco Pais, Tocha, Portugal; ^4^ Faculty of Medicine, University Coimbra, Coimbra, Portugal

**Keywords:** education, rehabilitation, functioning, physical and rehabilitation medicine, health system, capacity building, international classification of functioning disability and health (IFC)

**Editorial on the Research Topic** Editorial: Pursuing quality education in Physical and Rehabilitation Medicine

## Introduction

What is the value of education in Physical and Rehabilitation Medicine (PRM) and why bothering about it would strengthen rehabilitation in health systems and eventually improve health care? The collection of articles in the Special Issue about Pursuing quality education in Physical and Rehabilitation Medicine addresses these fundamentally important questions through different approaches:

- Launching a call for optimizing education in PRM as a tool for relieving the burden of disability in low-income countries (Cannata et al., Tannor et al.);
- Providing examples of the current and near-future approaches to PRM education, (Asami, Herrera-Ligero et al., Leochico et al.);
- Highlighting strategies to ensure the highest level of postgraduate training as a mean to support high-quality interventions of PRM physicians in disability and health care (Brown et al., Posada-Borrero et al., Scheel-Sailer et al.);
- Describing how the competency-based education for PRM trainees has changed during the COVID-19 pandemic (Leochico et al.); and
- Emphasizing the importance of teaching the basics of scientific research to postgraduate PRM trainees to prepare them to choose evidence-based treatments, promote scientific research in PRM and eventually improve management (Thibaut et al.).

## Why bother about education in PRM?

PRM is defined as the “medicine of functioning”. This is a key concept to understanding the value of this medical specialty, linking its scope to the intrinsic value of rehabilitation. Rehabilitation is a person-centred process including interventions designed to optimize functioning in individuals with health conditions or impairments in interaction with their environment (1); as such, it is an essential health strategy for increasing health and well-being, improving quality of life, delaying the need for long-term care and empowering persons to achieve their full potential and participate in society (2). Lack of access to rehabilitation may expose persons with rehabilitation needs to higher risks of marginalisation, poverty, vulnerability, complications, and comorbidities, adversely impacting their function, participation, and inclusion in society (3).

On May 27th 2023, the 76th World Health Assembly (WHA) adopted a landmark resolution on “Strengthening Rehabilitation in Health Systems”, taking a historic step towards acknowledging that rehabilitation is an essential element of universal health coverage, while admitting that worldwide rehabilitation needs are largely unmet, and more than 50% of people miss the rehabilitation services they require in many low and middle-income countries (LMIC).

This inadequacy is the outcome of several interacting factors:

a) Poor consideration of functioning as the third indicator of health, complementing morbidity and mortality, and, consequently, poor integration of its assessment in the health information system and poor attention to functioning by policymakers, when setting health priorities and allocating resources;
b) Limited exposure of medical students to training in the care of people with disabilities; this issue is of particular relevance when we consider the emphasis given by the World Health Organization (WHO) on the need to integrate rehabilitation services within all health system levels, including primary health care (4)
c) Lack of awareness among healthcare providers of the relevance of rehabilitation across the life course and for a wide range of health conditions;
d) Poor promotion of academic capacity in PRM worldwide;
e) Poor awareness that quality education and training of rehabilitation professionals (including PRM physicians) is an investment in the health of populations;
f) Insufficient workforce and equipment to respond to the increase in rehabilitation needs created by the progressive population aging combined with the impressive expansion of chronic disorders.

The Rehabilitation 2030 initiative introduced a “call for action”, gathering stakeholders towards coordinated global actions to scale up rehabilitation (5). Among the 10 priority areas for action, the need for:

• Creating strong leadership and political support for rehabilitation at different levels,
• Developing a strong multidisciplinary rehabilitation workforce,
• Promoting rehabilitation concepts across all health workforce education, and
• Building research capacity to expand the availability of robust evidence for rehabilitation

calls for emphasising the pivotal role of PRM physicians and harmonising post-graduate education in PRM.

To accomplish their role of “medical specialists of functioning”, the PRM physicians are called to plan the rehabilitation process, tailoring it to the individual health needs (6). With more than 2.4 billion individuals worldwide experiencing a vast range of physical and mental health conditions (permanent or temporary)—and potentially benefitting from rehabilitation (7), the PRM physicians are expected to develop an equally wide range of competencies and skillsets. Moreover, the strong collaborative association with other rehabilitation professionals calls for the development of good leadership, management and communication skills.

Education in PRM is also required at the undergraduate level, in medical schools. Virtually all physicians will encounter people with disabilities in their clinical practice across various pathologic conditions. However, studies demonstrate that people with disabilities are inadequately referred for rehabilitation even in developed countries. A systematic analysis of academic medical institution education offers in the United States reported that the undergraduate medical education system does not adequately train students to provide care for people with disabilities. The most common reason for not delivering a disability awareness curriculum was that no one advocated for its inclusion (8).

Advocacy is the mission of the World Rehabilitation Alliance (WRA), a WHO global network of stakeholders, committed to supporting the implementation of the Rehabilitation 2030 Initiative, by increasing the awareness and demand for rehabilitation, and, especially in LMIC, driving investment in quality rehabilitation education and training, and expanding the integration of the rehabilitation workforce into all levels of care and practice settings (9).

## Pursuing quality education in PRM: where are we?

In the European Union, harmonising staff education at the undergraduate and postgraduate levels is a mandatory element for ensuring the highest standard of rehabilitation care across different countries (10).

To support the widespread adoption of standards in PRM education, and overcome the current discrepancies in the duration and contents of PRM training throughout Europe, the European Board for PRM has released the European training requirements (ETR) for PRM education, that detail the theoretical knowledge (learning outcomes) and the core competencies to be achieved at the end of training (training outcomes), in preparation for the independent practice of PRM (11).

At the world level, the Education Committee of the International Society of PRM (ISPRM) released the first version of the recommended Core Curriculum and Competency, in 2019 (12) with the goal of providing a set of fundamental practical knowledge requirements and competencies expected in the professional practice of PRM. The document considers the variability in practice and resource availability in each geographic location, so the emphasis is placed on basic concepts and principles of PRM, with the addition of some topics/conditions, to serve as a guide for training programs.

Developing competency-based education can represent a powerful mechanism to align education and training with health system priorities. This holds particular value for resource-limited countries, where the knowledge and skills of rehabilitation doctors need to reflect not only the population's health profile but also the strengths and weaknesses (e.g., workforce gaps and maldistributions) of the health system.

In the African Region, where there is a substantial lack of PRM physician workforce, as PRM training programs are missing, the International Rehabilitation Forum (IRF) developed and implemented a fellowship program to train physicians in rehabilitation medicine, in Ghana, Ethiopia and Cameroon (all LMICs in sub-Saharan Africa). Tannor et al. commented the IRF initiative shedding light on the ongoing challenges of inadequate availability of PRM trainers, logistics and services for hands-on experience, and funding. They also reported how it becomes especially difficult to set up PRM training programs in countries where not only PRM trainers but also allied rehabilitation professionals are missing.

## Conclusion

High-quality rehabilitation care represents a constitutive element of health systems worldwide. The harmonisation of staff education both at the undergraduate and postgraduate level is a key element for ensuring the highest standard of rehabilitation care. International bodies, like the UEMS Board for Physical and Rehabilitation Medicine (PRM) or the ISPRM, have already delivered regulatory documents setting standards in postgraduate PRM education. Implementing such rules is to be validated worldwide with special attention to low and middle-income countries.
